# Supplementary material for: Structural insight into the human SID1 transmembrane family member 2 reveals its lipid hydrolytic activity
Source: Nat Commun. 2023 Jun 15;14:3568. doi: 10.1038/s41467-023-39335-2 (PMC10272179; doi:10.1038/s41467-023-39335-2)
Supplement: Supplementary file 1 — Supplementary Information [file 41467_2023_39335_MOESM1_ESM.pdf]

Supplementary Information for

Structural insight into the human SID1 transmembrane family member 2 reveals its lipid hydrolytic activity

Dandan Qian[1#], Ye Cong[2,3,4,5#], Runhao Wang[1], Quan Chen[1*], Chuangye Yan[2,3,4,5*], Deshun Gong[1*]

[1]State Key Laboratory of Medicinal Chemical Biology and College of Life Sciences, Nankai University, Tianjin 300350, China.

[2]School of Life Sciences, Tsinghua University, Beijing, 100084, China.
[3]Tsinghua-Peking Joint Center for Life Sciences, Tsinghua University, Beijing, 100084, China.
[4]Beijing Frontier Research Center for Biological Structure, Beijing Advanced Innovation Center for Structural Biology, Tsinghua University, Beijing, 100084, China.
[5]State Key Laboratory of Membrane Biology, Tsinghua University, Beijing, 100084, China.

* To whom correspondence should be addressed:
chenq@nankai.edu.cn; yancy2019@tsinghua.edu.cn; gongds@nankai.edu.cn.

This PDF file includes

- 14 Supplementary Figures
- 1 Supplementary Table
- The source data underlying Supplementary Fig. 2a and 2b.

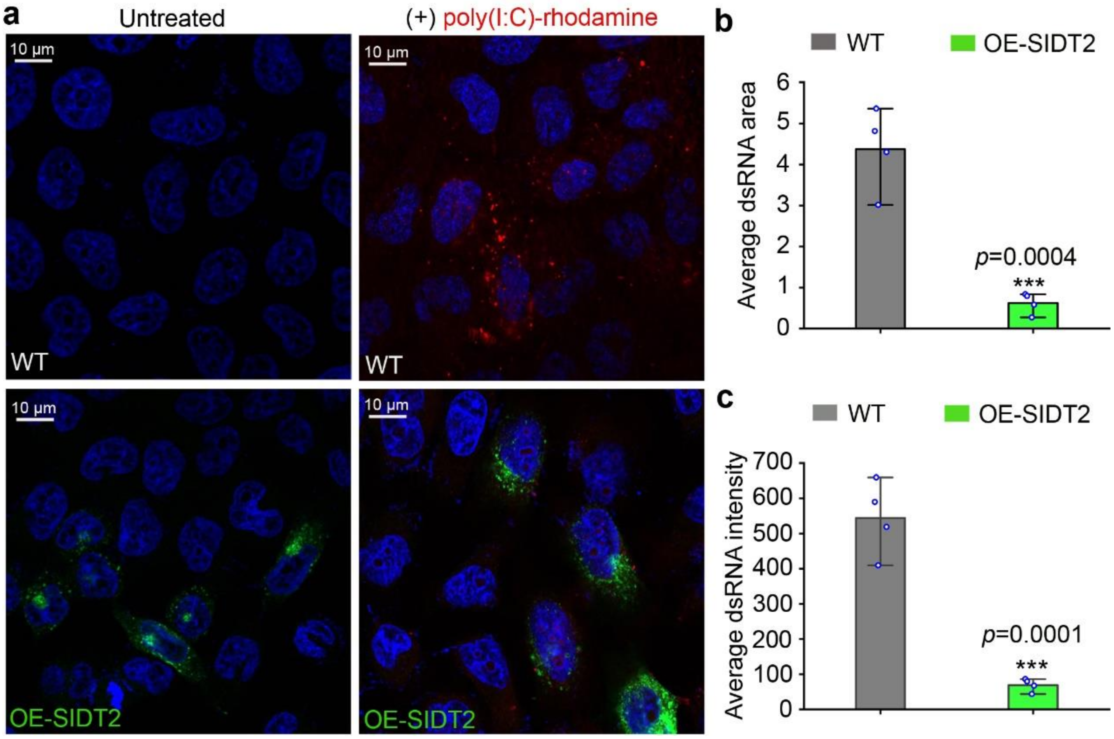

Supplementary Fig. 1 | Overexpression of SIDT2 changes the intracellular localization of poly(I:C)-rhodamine. a, Wild-type Hela cells (WT) and the cells that overexpression of SIDT2 (OE-SIDT2) were treated with poly(I:C)-rhodamine for 24h and imaged by confocal microscopy. The dsRNA in WT was significantly more punctate than that of OE-SIDT2. red = poly(I:C), blue = DAPI, green=SIDT2. **b, c,** The average dsRNA area and intensity were quantified using FIJI software. Data are plotted as mean ± s.d. and four representative fields of view each containing 10-20 cells were analyzed per condition (n=4). Asterisks indicate statistical significance by two-tailed Student's t-tests (***$p < 0.001$). 95% confidence interval of the difference in **b**: from 2.4869134 to 5.0223066. 95% confidence interval of the difference in **c**: from 342.9804293 to 608.0056907. Source data are provided as a Source Data file.

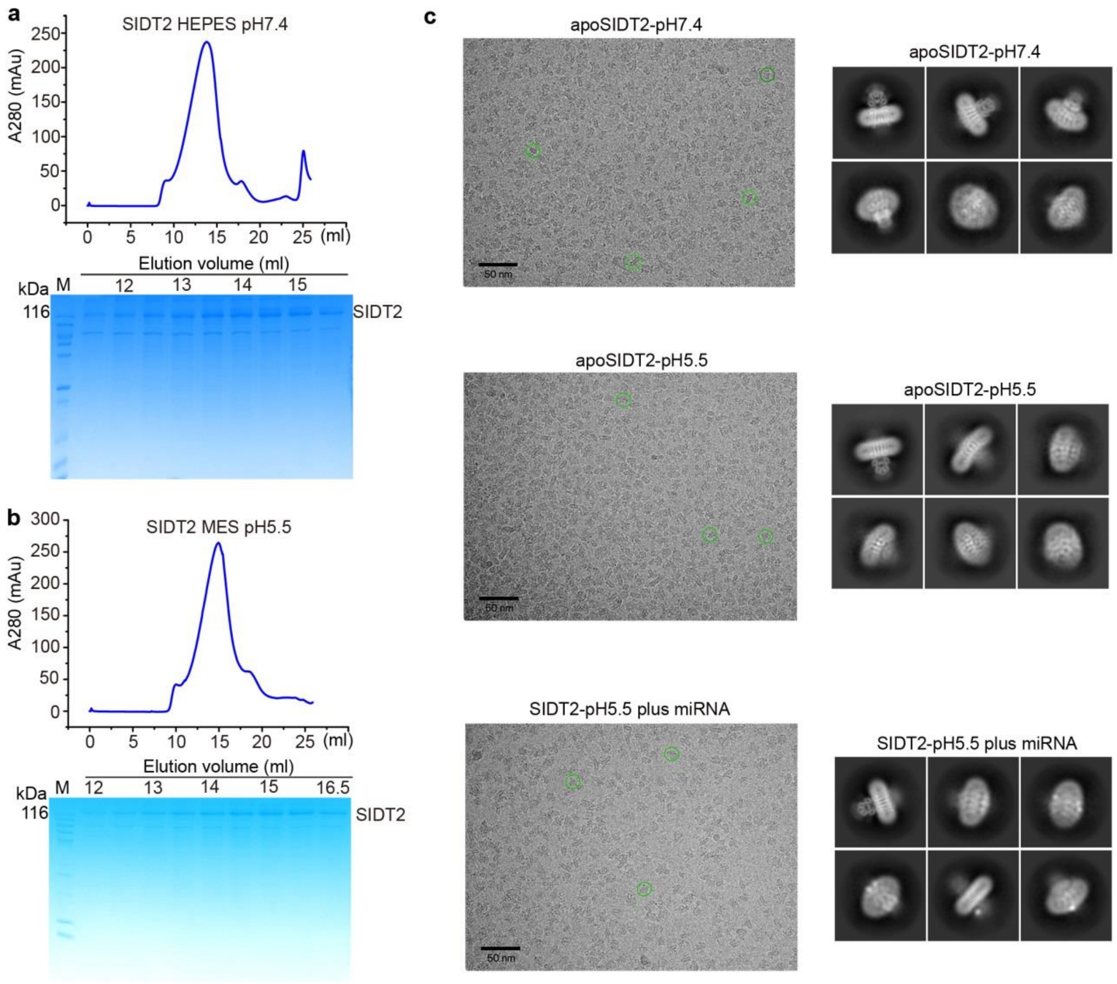

Supplementary Fig. 2 | Protein purification and cryo-EM analysis of human SIDT2. a,b, The SIDT2 protein was subjected to size exclusion chromatography (SEC) in different pH conditions. Peak fractions were visualized by Coomassie blue staining. kDa, kilodaltons. M, marker. **c,** Representative cryo-EM micrographs and 2D class averages of SIDT2 under the three conditions. Experiment was repeated independently more than three times with similar results. Source data are provided as a Source Data file.

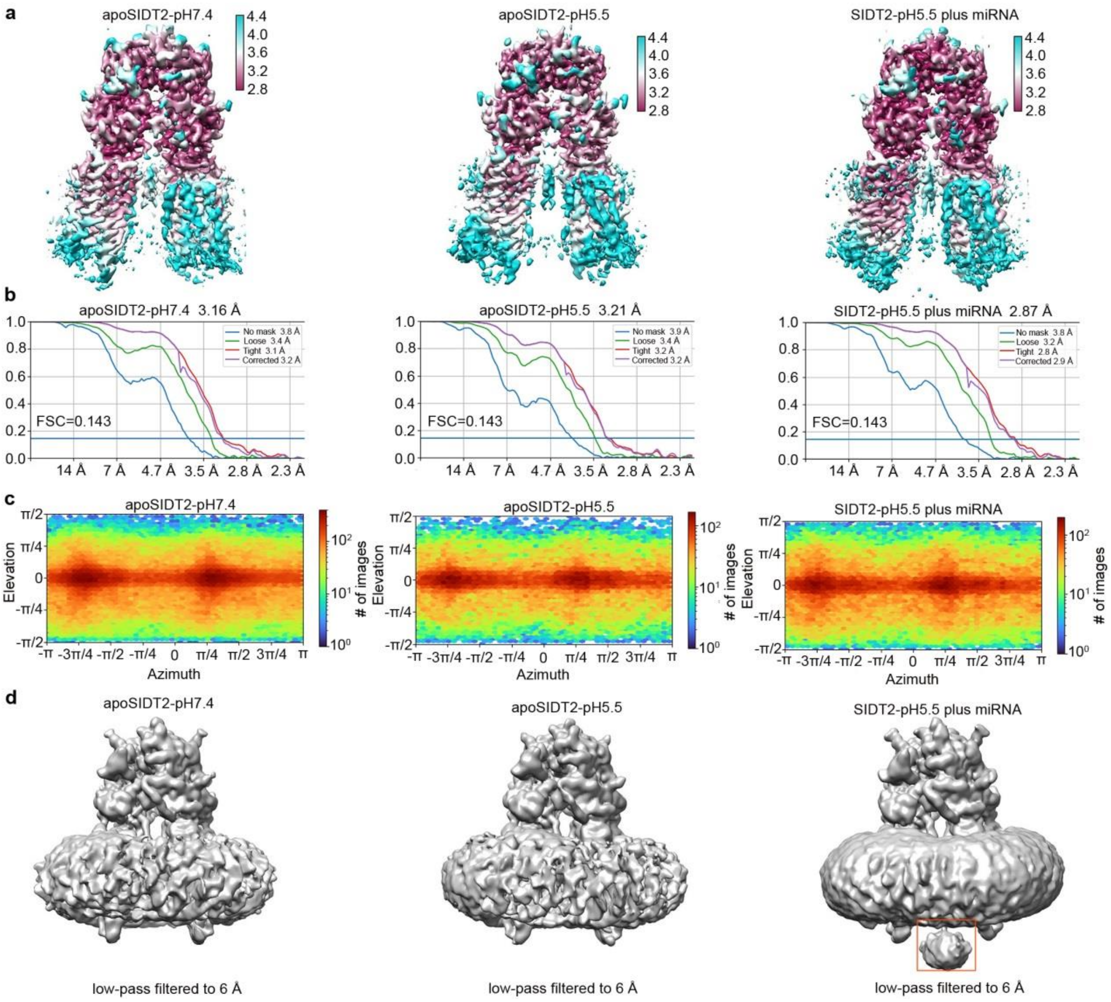

Supplementary Fig. 3 | Local resolution maps, resolution estimation, angular distribution, and low-pass filtered maps of the three reconstructions. a, The local resolution maps of the three reconstructions. **b,** Gold-standard Fourier shell correlation curves for electron microscopy maps of the three datasets. **c,** Angular distribution of the particles used for the final reconstructions. **d,** The low-pass filtered maps of the three reconstructions. An additional unmodeled density was observed on the cytoplasmic side in the map of SIDT2-pH 5.5 plus miRNA.

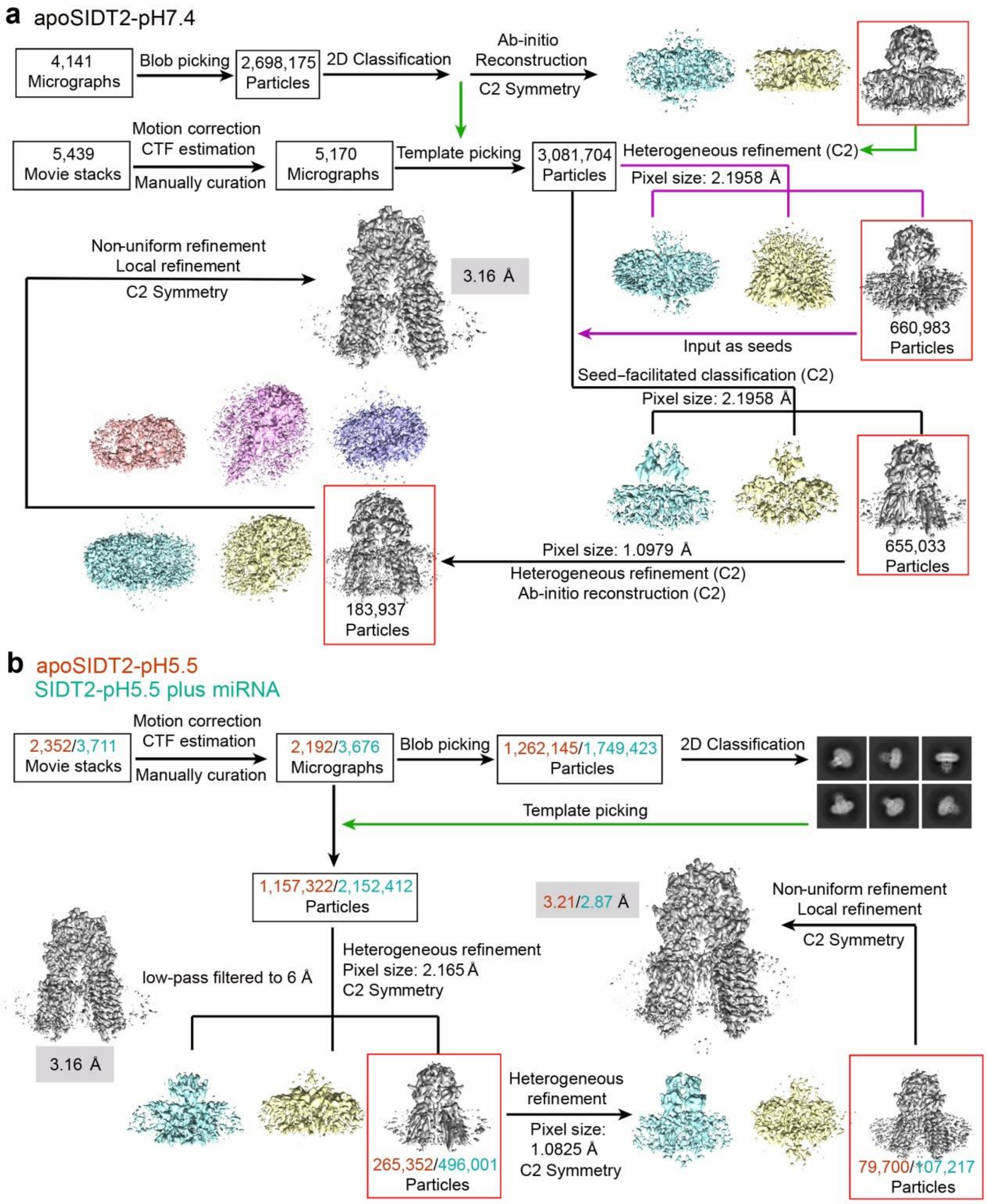

Supplementary Fig. 4 | Flowchart for cryo-EM data processing. Please refer to Methods for details.

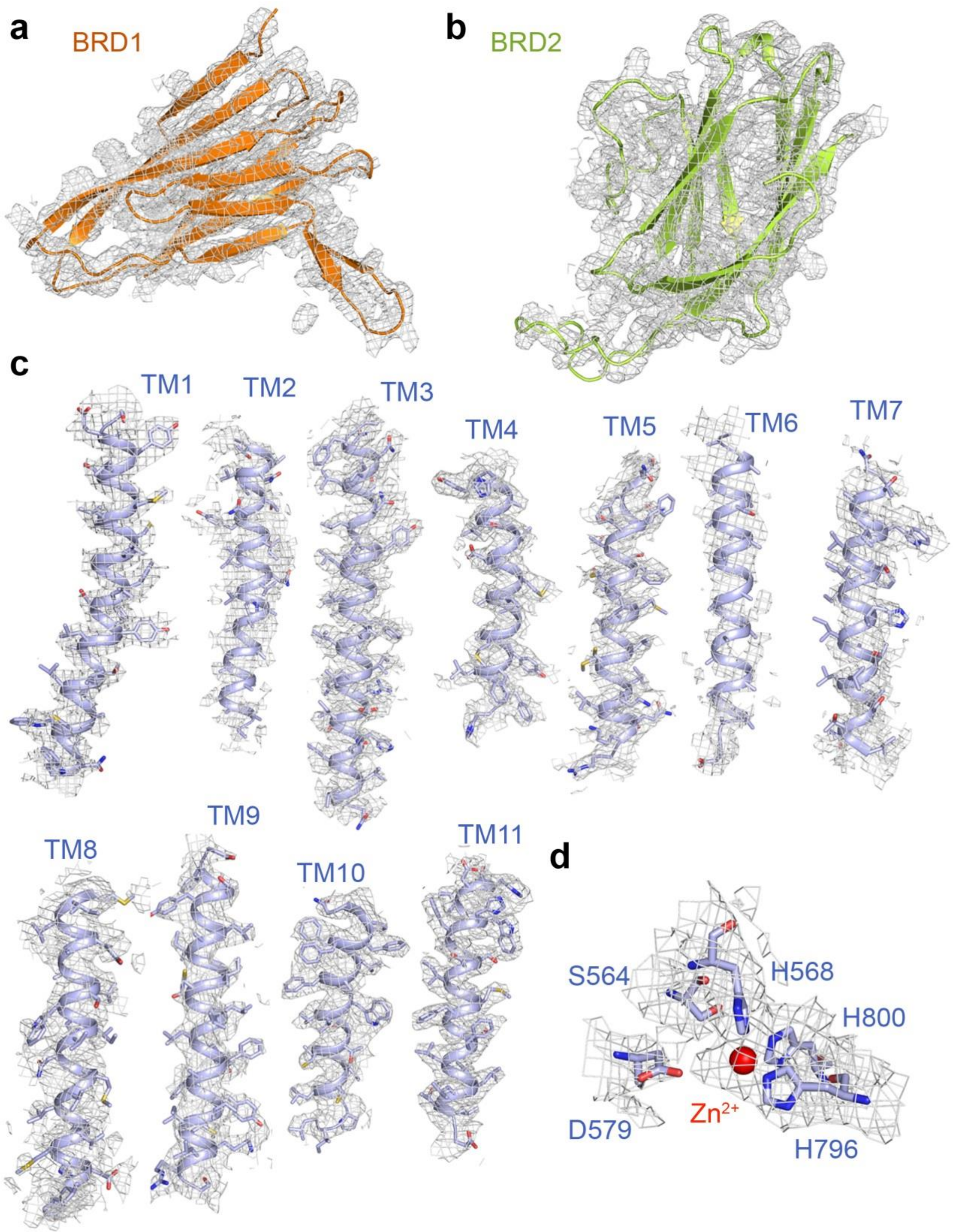

Supplementary Fig. 5 | Representative local EM maps of SIDT2. a, The EM map of the BRD1. **b,** The EM map of the BRD2. **c,** The EM maps of the eleven TM helices. **d,** The EM maps of the putative Zn^{2+}-binding site.

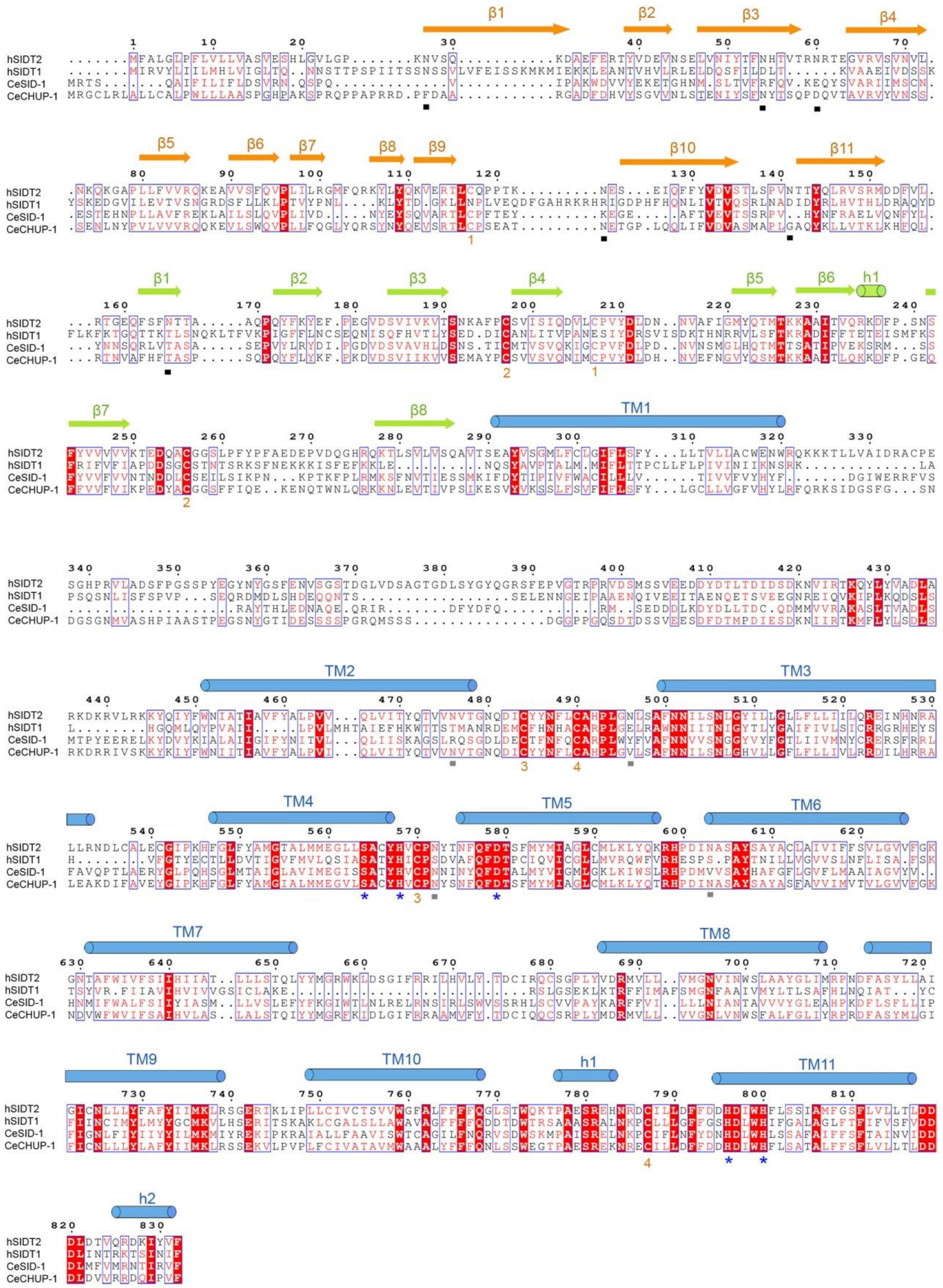

Supplementary Fig. 6 | Sequence alignment of SIDT2 with its homologues. The sequences were aligned using ClustalW. The secondary structural elements of SIDT2 are indicated above the sequence alignment and domains are colored the same as the structures shown in the main text figures. h, *homo sapiens*; Ce, *C. elegans*. The orange numbers indicate the four pairs of disulfide bonds. The blue stars indicate the putative catalytic Zn^{2+}-binding site. The grey boxes indicate the glycosylation sites.

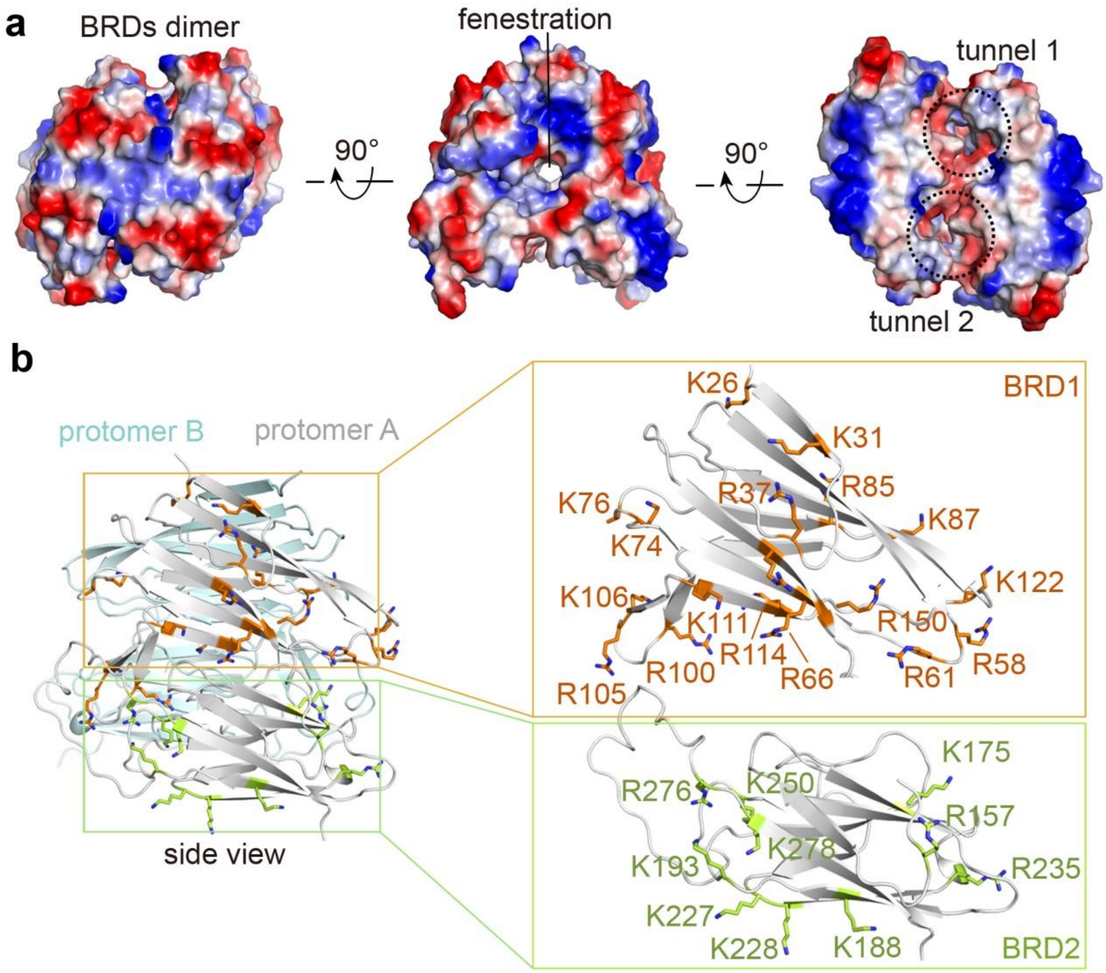

Supplementary Fig. 7 | Structural feature of BRDs. a, The electrostatic potential surface of BRDs dimer. A fenestration was observed in the dimer interface. Two separated tunnels are formed from the fenestration to the bottom of the BRD2 dimer interface. **b,** Both the BRD1 and BRD2 are rich in positively charged residues.

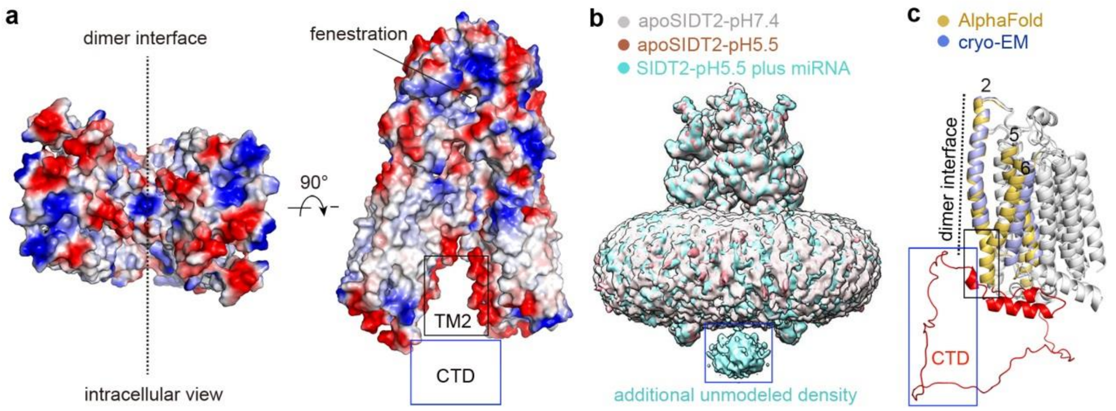

Supplementary Fig. 8 | The TMD exhibits no clear characteristic of a channel protein. a, No clear nucleic acid or ion conducting pathway was found in the TMD, which is shown as an electrostatic potential surface. The black box indicates the unresolved portion of TM2, and the blue box indicates the unresolved CTD. **b,** Comparison of the three structures captured under three different conditions. No conformational change was found among these three structures. An additional area of density located in the CTD is observed in the map of SIDT2-pH5.5 plus miRNA, blocking the cytoplasmic entry point in the dimer interface. **c,** Comparison of the cryo-EM structure of SIDT2 with that of AF-SIDT2 indicated the location of the invisible CTD. A portion of the CTD was predicted by AlphaFold and is located in the dimer interface, suggesting that the additional area of density is likely a portion of the CTD.

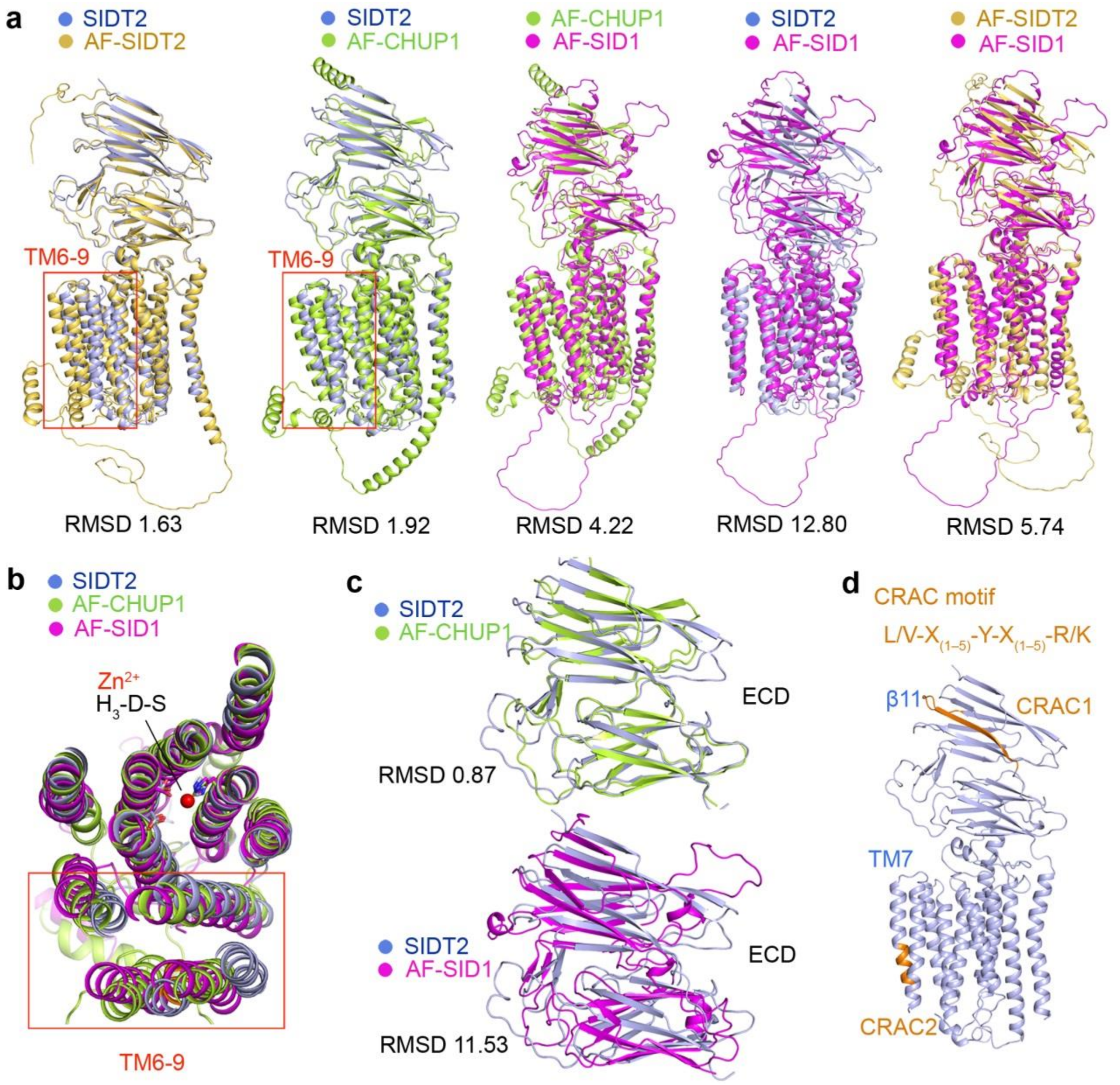

Supplementary Fig. 9 | Pairwise comparison of the structures of SIDT2, AF-SIDT2, AF-SID1, and AF-CHUP1 and locations of the two CRACs. a, The structure of SIDT2 protomer is strikingly similar to that of CHUP1. All the structures were superimposed relative to the intact structures. TM6-9 of SIDT2 underwent obvious conformational changes compared to those of AF-SIDT2 and AF-CHUP1. RMSD, root-mean-square deviation. AF, Alphafold. **b,** TM6-9s displayed different conformations, whereas the other TMs adopted nearly identical conformations. All the structures were superimposed relative to the Zn^{2+}-binding site. **c,** The ECD of SIDT2 showed a nearly identical conformation to that of CHUP1, whereas it underwent marked conformational changes compared to that of SID1. All the structures were superimposed relative to the ECD. **d,** Locations of the two CRACs on the SIDT2.

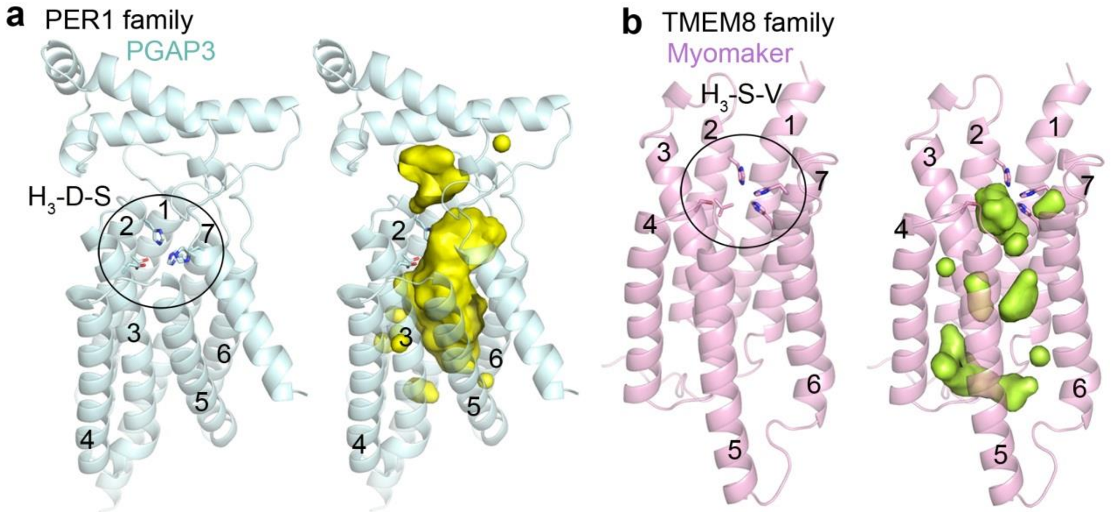

Supplementary Fig. 10 | Structural features of the TMDs of the AF-PGAP3 in Per1 family and the AF-myomaker in the TMEM8 family. a, The highly conserved catalytic Zn^{2+}-binding site in a large cavity within the TMD was found in the AF-PGAP3 (Post-GPI Attachment To Proteins Phospholipase 3) structure. **b,** Two mutations occur in the Zn^{2+}-binding site of myomaker, the large cavity was lost in the TMD.

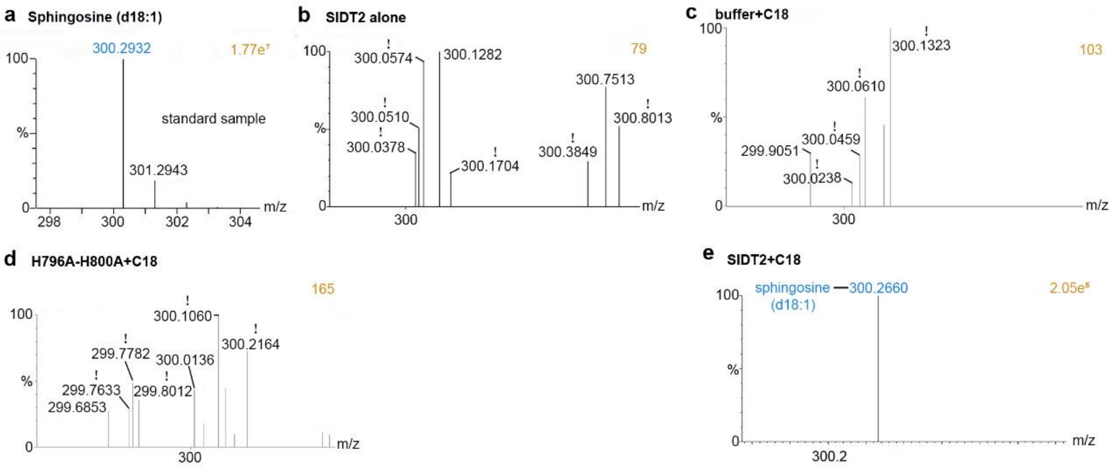

Supplementary Fig. 11 | Sphingosine detected by LC–MS analysis revealed SIDT2 ceramidase activity. a, Representative mass spectrum for the extracted ion peak of the d-erythro-sphingosine (d18:1) standard sample. **b,** Representative mass spectrum for the extracted ion peak of the SIDT2 alone sample. No signal for sphingosine (d18:1) was detected. **c,** Representative mass spectrum for the extracted ion peak of the reaction buffer plus ceramide (d18:1/18:0) (buffer+C18) sample. No signal for sphingosine (d18:1) was detected. **d,** Representative mass spectrum for the extracted ion peak of the H796A-H800A mutant plus ceramide (d18:1/18:0) (H796A-H800A+C18) sample. No signal for sphingosine (d18:1) was detected. **e,** Representative mass spectrum for the extracted ion peak of the wild-type SIDT2 plus ceramide (d18:1/18:0) (SIDT2+C18) sample. Strong signal for sphingosine (d18:1) was detected.

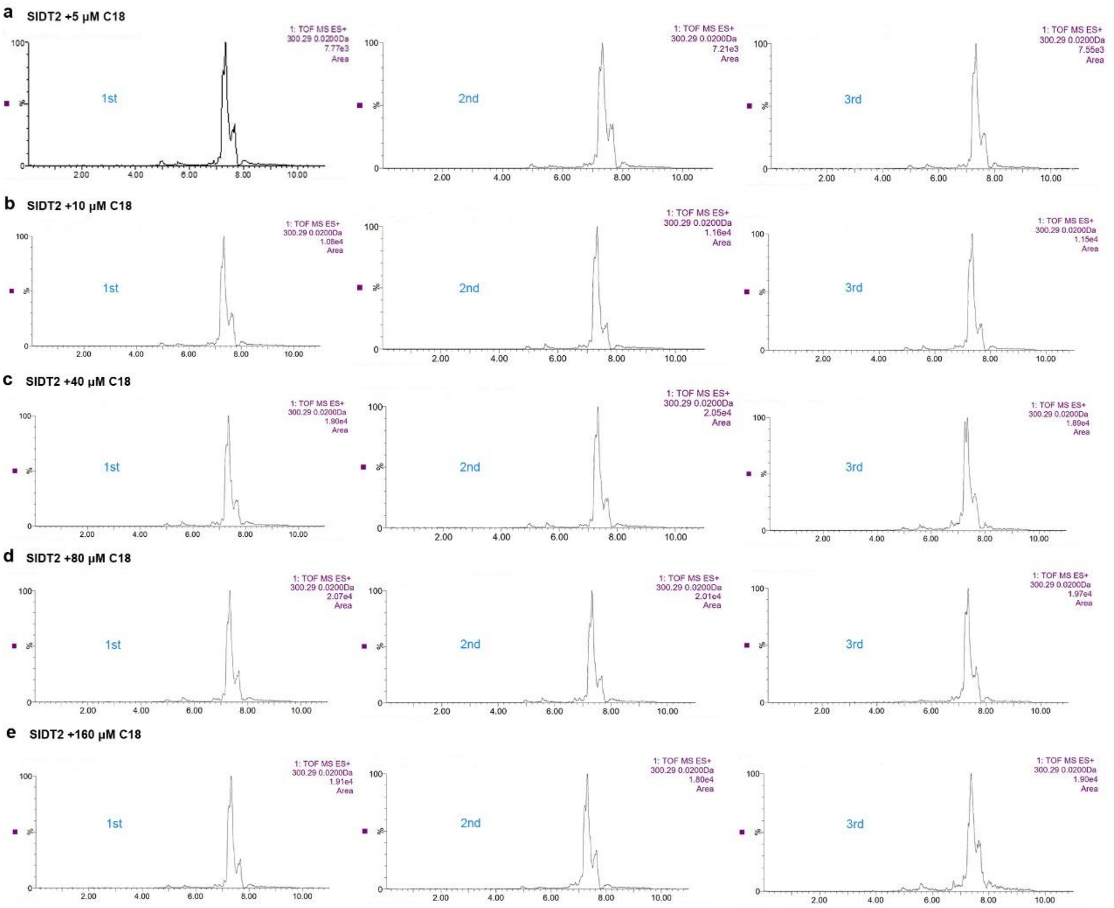

Supplementary Fig. 12 | Quantification of the ceramidase activity of SIDT2. a-e, Representative LC–MS analysis with an extracted ion chromatogram revealing the relative amounts of detected sphingosine (d18:1), which was generated from the enzymatic reaction between SIDT2 with increasing amounts of ceramide (d18:1/18:0) substrate (5, 10, 40, 80, 160 µM). Each experiment was performed in triplicate.

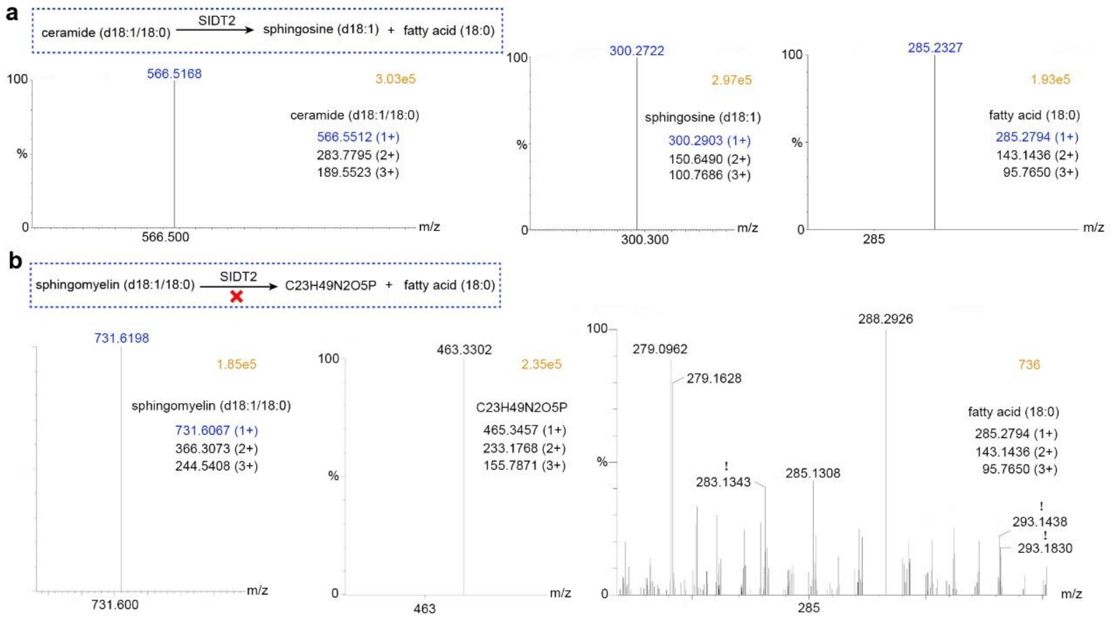

Supplementary Fig. 13 | The substrate specificity of SIDT2. a, SIDT2 can convert ceramide (d18:1/18:0) into sphingosine (d18:1) and fatty acid (18:0). Representative mass spectrum for the extracted ion peaks of ceramide (d18:1/18:0), sphingosine (d18:1), and atty acid (18:0). Strong signals were detected. **b,** SIDT2 failed to hydrolyze the sphingomyelin (d18:1/18:0). No signals for the products were detected.

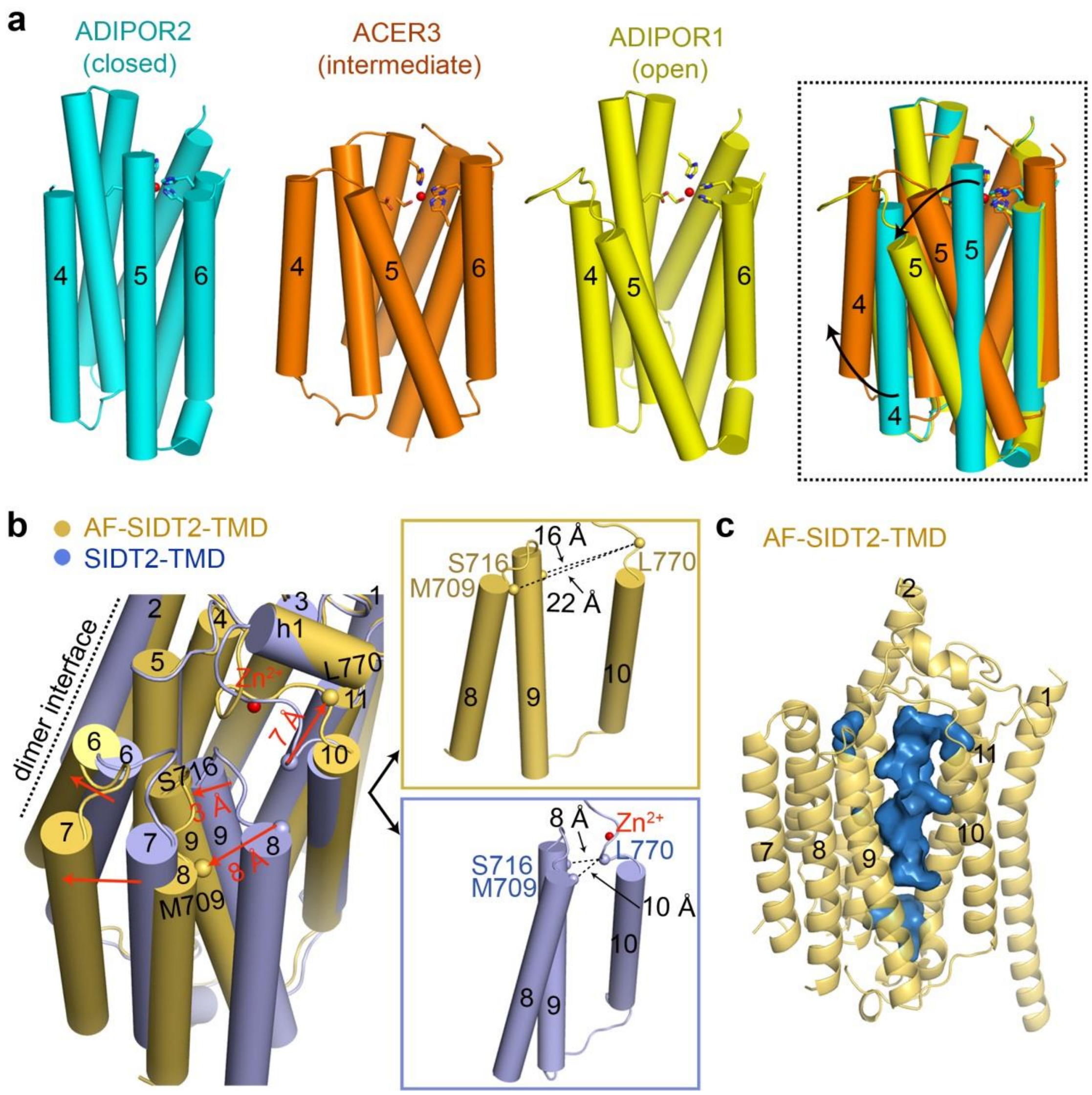

Supplementary Fig. 14 | Possible different steps in the Zn^{2+}-dependent intramembrane catalytic process. a, A closed ADIPOR2 structure bound an oleic acid, an intermediate ACER3 structure bound a monoolein, and an open ADIPOR1 structure without a ligand were captured. **b,** Superimposing the TMD of SITD2 with that of AF-SIDT2 revealed that TM6-9 and the loop between TM10 and helix 1 (h1) underwent dramatic conformational changes. **c,** A larger cavity was observed in the TMD of AF-SIDT2.

Supplementary Table 1: Cryo-EM data collection, refinement and validation statistics

	**apoSIDT2-pH7.4**	**apoSIDT2-pH5.5**	**SIDT2-pH5.5 plus miRNA**
**Data collection and processing**			
Magnification	64,000	81,000	81,000
Voltage (kV)	300	300	300
Electron exposure ($e^{-}/Å^2$)	50	50	50
Defocus range (μm)	-1.3 to -1.8	-1.3 to -1.8	-1.3 to -1.8
Pixel size (Å)	1.0979	1.0825	1.0825
Symmetry imposed	C2	C2	C2
Movies	5,170	2,192	3,676
Final number of particles (no.)	183,937	79,700	107,217
Map resolution (Å)	3.16	3.21	2.87
FSC threshold	0.143	0.143	0.143
**Refinement**			
Model composition			
Non-hydrogen atoms	9,926	10,092	10,266
Protein residues	1,238	1,274	1,300
Ligands	16	16	18
B factors ($Å^2$)			
Protein	75.1	85.6	83.8
Ligand	76.5	84.1	86.7
R.m.s. deviations			
Bond lengths (Å)	0.004	0.005	0.007
Bond angles (°)	0.632	0.675	1.168
Validation			
MolProbity score	1.94	2.00	1.98
Clashscore	11.8	15.6	12.4
Poor rotamers (%)	0.0	0.0	0.57
Ramachandran plot			
Favored (%)	94.8	95.7	94.6
Allowed (%)	5.2	4.3	5.4
Disallowed (%)	0.0	0.0	0.0

Source Data for the gel in Supplementary Fig. 2a

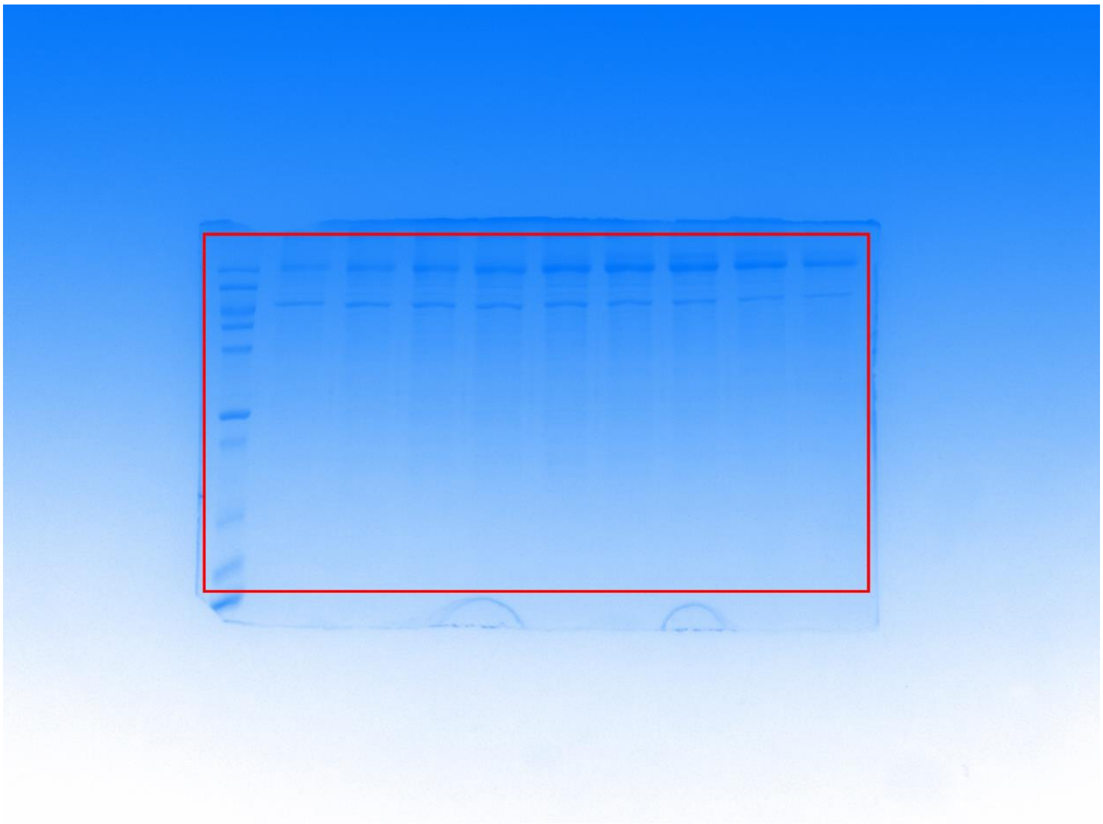

Uncropped scan of the gel in Supplementary Fig. 2a

Source Data for the gel in Supplementary Fig. 2b

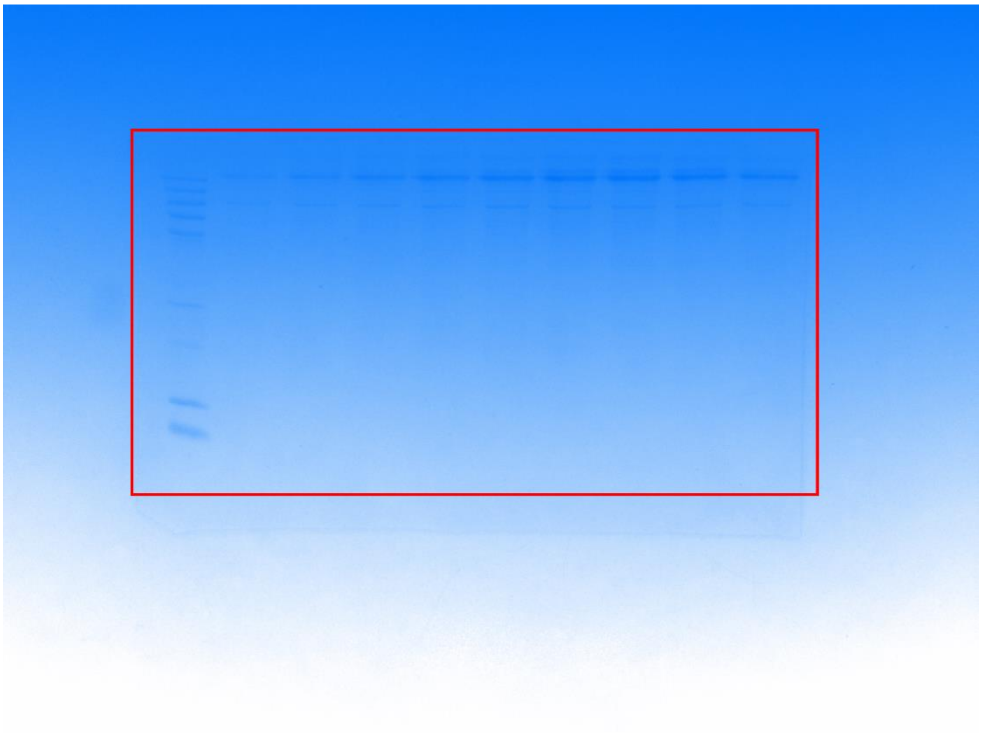

Uncropped scan of the gel in Supplementary Fig. 2b
