# Supplementary material for: Structural insight into the human SID1 transmembrane family member 2 reveals its lipid hydrolytic activity
Source: Nat Commun. 2023 Jun 15;14:3568. doi: 10.1038/s41467-023-39335-2 (PMC10272179; doi:10.1038/s41467-023-39335-2)
Supplement: Supplementary file 3 — Description of Additional Supplementary Files [file 41467_2023_39335_MOESM3_ESM.pdf]

File name: Supplementary Data 1

Description: Quantification of the ceramidase activity of SIDT2. LC–MS analysis of the relative amounts of detected sphingosine (d18:1). Each experiment was performed in triplicate.
