# Supplementary material for: Structural insight into the human SID1 transmembrane family member 2 reveals its lipid hydrolytic activity
Source: Nat Commun. 2023 Jun 15;14:3568. doi: 10.1038/s41467-023-39335-2 (PMC10272179; doi:10.1038/s41467-023-39335-2)
Supplement: Supplementary file 5 — Reporting Summary [file 41467_2023_39335_MOESM5_ESM.pdf]

nature portfolio

Corresponding author(s): Quan Chen
Chuangye Yan
Deshun Gong

Last updated by author(s): May 29, 2023

Reporting Summary

Nature Portfolio wishes to improve the reproducibility of the work that we publish. This form provides structure for consistency and transparency in reporting. For further information on Nature Portfolio policies, see our Editorial Policies and the Editorial Policy Checklist.

Statistics

For all statistical analyses, confirm that the following items are present in the figure legend, table legend, main text, or Methods section.

n/a	Confirmed	
☐	☒	The exact sample size (*n*) for each experimental group/condition, given as a discrete number and unit of measurement
☐	☒	A statement on whether measurements were taken from distinct samples or whether the same sample was measured repeatedly
☐	☒	The statistical test(s) used AND whether they are one- or two-sided<br>*Only common tests should be described solely by name; describe more complex techniques in the Methods section.*
☒	☐	A description of all covariates tested
☒	☐	A description of any assumptions or corrections, such as tests of normality and adjustment for multiple comparisons
☐	☒	A full description of the statistical parameters including central tendency (e.g. means) or other basic estimates (e.g. regression coefficient) AND variation (e.g. standard deviation) or associated estimates of uncertainty (e.g. confidence intervals)
☐	☒	For null hypothesis testing, the test statistic (e.g. *F*, *t*, *r*) with confidence intervals, effect sizes, degrees of freedom and *P* value noted<br>*Give P values as exact values whenever suitable.*
☒	☐	For Bayesian analysis, information on the choice of priors and Markov chain Monte Carlo settings
☒	☐	For hierarchical and complex designs, identification of the appropriate level for tests and full reporting of outcomes
☒	☐	Estimates of effect sizes (e.g. Cohen's *d*, Pearson's *r*), indicating how they were calculated

Our web collection on statistics for biologists contains articles on many of the points above.

Software and code

Policy information about availability of computer code

Data collection	AutoEMation v2, Confocal microscope Zeiss LSM800 with Airscan, Waters ACQUITY UPLC I-Class.
Data analysis	MotionCor2 v1, cryoSPARC v3.2, COOT v0.9.3, PHENIX v1.18, UCSF ChimeraX v1.2, Chimera v1.11, Origin 2022, Pymol v1.8.0.4, FIJI win 64-bit, predicted structures were downloaded from the AlphaFold protein structure database (https://www.alphafold.ebi.ac.uk/), GraphPad (https://www.graphpad.com/quickcalcs/ttest2/).

For manuscripts utilizing custom algorithms or software that are central to the research but not yet described in published literature, software must be made available to editors and reviewers. We strongly encourage code deposition in a community repository (e.g. GitHub). See the Nature Portfolio guidelines for submitting code & software for further information.

Data

Policy information about availability of data

All manuscripts must include a data availability statement. This statement should provide the following information, where applicable:

- Accession codes, unique identifiers, or web links for publicly available datasets
- A description of any restrictions on data availability
- For clinical datasets or third party data, please ensure that the statement adheres to our policy

The atomic coordinates of apoSIDT2-pH 7.4, apoSIDT2-pH 5.5, and SIDT2-pH 5.5 plus miRNA have been deposited in the PDB (http://www.rcsb.org) under the accession code 7Y63 [https://www.rcsb.org/structure/unreleased/7Y63], 7Y69 [https://www.rcsb.org/structure/unreleased/7Y69], and 7Y68 [https://www.rcsb.org/

structure/unreleased/7Y68], respectively; The electron microscopy density maps of these three structures have been deposited in the Electron Microscopy Data Bank (EMDB https://www.ebi.ac.uk/pdbe/emdb/) under the accession code EMD-33632 [https://www.ebi.ac.uk/emdb/EMD-33632], EMDB: EMD-33638 [https://www.ebi.ac.uk/emdb/EMD-33638], and EMD-33637 [https://www.ebi.ac.uk/emdb/EMD-33637], respectively. The source data underlying Fig. 5a, 5b, and Supplementary Fig. 1b, 1c, 2a, and 2b are provided as a Source Data file. Source data are provided with this paper.

Human research participants

Policy information about studies involving human research participants and Sex and Gender in Research.

Reporting on sex and gender	N/A
Population characteristics	N/A
Recruitment	N/A
Ethics oversight	N/A

Note that full information on the approval of the study protocol must also be provided in the manuscript.

Field-specific reporting

Please select the one below that is the best fit for your research. If you are not sure, read the appropriate sections before making your selection.

☒ Life sciences ☐ Behavioural & social sciences ☐ Ecological, evolutionary & environmental sciences

For a reference copy of the document with all sections, see nature.com/documents/nr-reporting-summary-flat.pdf

Life sciences study design

All studies must disclose on these points even when the disclosure is negative.

Sample size	For cryo-EM studies, sample size was determined by availability of microscope. For all the functional assays, no statistical methods were used to predetermine sample size. The sufficient sample size were chosen for statistical analysis.
Data exclusions	No data were excluded from the analysis.
Replication	All the functional assay were conducted at least three independent experiments with technical repeats. All attempts at replication were successful.
Randomization	Randomization is not relevant to this study, since no experimental group was assigned in all experiments.
Blinding	Blinding is not applicable to this study, since neither structural nor functional experiments included subjective assignments.

Reporting for specific materials, systems and methods

We require information from authors about some types of materials, experimental systems and methods used in many studies. Here, indicate whether each material, system or method listed is relevant to your study. If you are not sure if a list item applies to your research, read the appropriate section before selecting a response.

Materials & experimental systems

n/a	Involved in the study	
☐	☒	Antibodies
☐	☒	Eukaryotic cell lines
☒	☐	Palaeontology and archaeology
☒	☐	Animals and other organisms
☒	☐	Clinical data
☒	☐	Dual use research of concern

Methods

n/a	Involved in the study	
☒	☐	ChIP-seq
☒	☐	Flow cytometry
☒	☐	MRI-based neuroimaging

Antibodies

Antibodies used	Flag-M2 (1:1000, Sigma, Cat. No F1804), anti-mouse Alexa-488 secondary antibody (1:1000, ThermoFisher, Cat. No A32723)

March 2021

Validation	All antibodies used in this study are commercially available and have been validated by the manufactures accordingly.<br>FLAG-M2: (1:1000, Sigma, Cat. No F1804), Host: mouse. antibody website: https://www.sigmaaldrich.cn/CN/en/product/sigma/f1804.<br>anti-mouse Alexa-488 secondary antibody (1:1000, ThermoFisher, Cat. No A32723), Host: goat. antibody website: https://www.thermofisher.cn/cn/zh/antibody/product/Goat-anti-Mouse-IgG-H-L-Highly-Cross-Adsorbed-Secondary-Antibody-Polyclonal/A32723.

Eukaryotic cell lines

Policy information about cell lines and Sex and Gender in Research

Cell line source(s)	HEK293F cell line was originally obtained from Invitrogen. Hela cell line was obtained from ATCC.
Authentication	Authentication was done by the manufactures by standard STR profiling analysis.
Mycoplasma contamination	All Cell lines tested are negative for mycoplasma contamination.
Commonly misidentified lines (See ICLAC register)	No commonly misidentified lines was used.
